# Massachusetts

**Published:** 1895-08

**Authors:** 


### MASSACHUSETTS.

The bill to compel registration of all veterinarians in Massachusetts has failed to become a law, largely owing to a difference of opinion among certain veterinarians as to a certain feature of the law, requiring a ten-years' qualification of actual practice to entitle the practitioner to the right of appointment on the Board of Registration. As this seemed to be the unanimous sentiment of the Massachusetts State Veterinary Association, it seems proper that this point, which is an important one, should have been maintained in the law. Veterinarians in every State must learn the lesson that the majority's decision must prevail if they expect to obtain recognition in legislative halls, for if difference of opinion exist among them these must be settled before an appeal is made before such tribunals.

